# Effects of SGLT2 inhibitors on hepatic fibrosis and steatosis: A systematic review and meta-analysis

**DOI:** 10.3389/fendo.2023.1144838

**Published:** 2023-03-01

**Authors:** Peipei Zhou, Ying Tan, Zhenning Hao, Weilong Xu, Xiqiao Zhou, Jiangyi Yu

**Affiliations:** ^1^ Department of Endocrinology, Jiangsu Province Hospital of Chinese Medicine, Affiliated Hospital of Nanjing University of Chinese Medicine, Nanjing, China; ^2^ The First Clinical Medical College, Nanjing University of Chinese Medicine, Nanjing, China

**Keywords:** sodium-glucose cotransporter 2 inhibitors, liver fibrosis, hepatic steatosis, systematic review, meta-analysis

## Abstract

**Objective:**

Clinical trials have shown that sodium-glucose cotransporter 2 inhibitors (SGLT2i) are closely associated with hepatic fibrosis and steatosis by FibroScan. This paper aimed at evaluating the effects of SGLT2i on hepatic fibrosis and steatosis, which are presented as liver stiffness measurement (LSM) and controlled attenuation parameter (CAP).

**Methods:**

PubMed, Embase, Cochrane Library, Web of Science, China National Knowledge Infrastructure Database, China Science and Technology Journal Database, and Wanfang Database were searched for randomized clinical trials from database establishment to 30 November 2022 with no language restrictions. The risk of bias was evaluated by Collaboration Handbook. Software Stata 17 and Review Manager (version 5.3) were used for meta-analysis.

**Results:**

A total of eight articles including 686 patients were included. Compared with the control group, our results showed that SGLT2i could lower levels of LSM [MD = −0.82, 95%CI (−1.38, −0.25), p = 0.005] and CAP [MD = −12.80, 95%CI (−20.57, −5.03), p = 0.001]. Further subgroup analyses indicated that SGLT2i presented more advantages on longer treatment duration and more serious steatosis in decreasing LSM. For CAP, SGLT2i exhibited a clear advantage in subgroup analyses of longer treatment duration, younger people, dapagliflozin, worse fibrosis, and steatosis.

**Conclusion:**

SGLT2i could reduce LSM and CAP in contrast to other antihyperglycemic drugs. However, the included studies are not definitive, and well-designed, more multi-centered, blinded randomized clinical trials are warranted to definitively establish reliable evidence.

## Introduction

Liver stiffness measurement (LSM) and controlled attenuation parameter (CAP), measured by FibroScan equipment, have been seen as a low-failure (3.2%), minimal-risk, high-reliability (>95%), and reproducible tool for quantifying hepatic fibrosis and steatosis with non-invasiveness in patients with non-alcoholic fatty liver disease (NAFLD) (1–3), respectively. Studies show that steatosis exacerbates LSM and fibrosis aggravates CAP, indicating that they enter a vicious circle (4–6). In addition, accumulating evidence supports that NAFLD and type 2 diabetes mellitus (T2DM) have become public health problems across the world; the overall prevalence of NAFLD in patients with T2DM is 55.5% and more than twofold higher than that in the general populations (7, 8). NAFLD and T2DM are universally acknowledged to frequently coexist and influence synergistically; as a result, they increase the risk of not only non-alcoholic steatohepatitis, advanced fibrosis, cirrhosis, and progression of T2DM but also cardiovascular diseases and other chronic complications of T2DM (9–11).

So far, except for nutritional modification and exercise alone, no pharmacotherapy is currently approved for hepatic fibrosis and steatosis irrespective of the presence of T2DM. Some antihyperglycemic drugs, such as pioglitazone, metformin, glucagon-like peptide 1 analog, and sodium-glucose cotransporter 2 inhibitors (SGLT2i), have shown some promising hepato-protective effects (12–15). Furthermore, it has been demonstrated that SGLT2i exert favorable effects apart from reducing glucotoxicity, such as reducing inflammation, oxidative stress, body weight, visceral adiposity, and arterial stiffness (16, 17). Earlier, several systematic reviews and meta-analyses had discussed the SGLT2i in patients with NAFLD paying attention to hepatic enzymes, liver fat content, and body composition, but there is no substantial evidence of hepatic fibrosis and steatosis (18–20). Thus, we conducted this meta-analysis to explore the effects of SGLT2i on hepatic fibrosis and steatosis, wherein LSM and CAP were the major outcome measures.

## Materials and methods

This study was designed in accordance with the Preferred Reporting Items for Systematic Reviews and Meta-Analyses (PRISMA) guidelines (21). In addition, this research was successfully registered at PROSPERO, the international prospective register of systematic reviews (registration number: CRD42022380160).

### Database and search strategies

Two researchers conducted a comprehensive literature search in the following seven electronic databases: PubMed, Embase, Cochrane Library, Web of Science, China National Knowledge Infrastructure Database (CNKI), China Science and Technology Journal Database (VIP), and Wanfang Database. The last search was conducted on 30 November 2022, with no language restrictions. The search terms were “sodium-glucose cotransporter 2 inhibitors”, “transient elastography”, “controlled attenuation parameter”, “liver stiffness”, “random”, and so on. Additionally, the reference lists of relevant reviews and included studies were also retrieved manually to obtain additional eligible literature. A detailed description of the search strategy in English databases is available in **Supplementary Material Table 1**.

### Inclusion criteria

Studies were selected if they were eligible for the following conditions: 1) participants: patients aged 18 years or older, regardless of sex and primary diseases, diagnosed with T2DM or NAFLD; 2) intervention: SGLT2i combined with the treatment of the control group; 3) comparator: the control group received placebo or no treatment or standard treatment according to the reality including adjusting diets and lifestyle, strengthening exercises, lowering hyperglycemia, controlling hypertension, and declining hyperlipidemia; 4) outcome: primary outcomes comprised of CAP or LSM that had to be recorded pre- and post-intervention or the difference between them; and 5) study design: randomized controlled trials (RCTs) of people irrespective of blinding, protocol or bias.

### Exclusion criteria

Studies were excluded if they were not eligible for the following conditions: 1) no SGLT2i in the intervention group; 2) SGLT2i combined with other treatments, which were not included in the control group; 3) observational studies, reviews, and experimentation on animals or cells; 4) non-RCTs; 5) duplication published later; and 6) incomplete papers.

### Data extraction

Two investigators independently retrieved the databases and filtered the studies according to the pre-designed inclusion and exclusion criteria. Then, EndNote 20 was utilized to eliminate duplication. In case of divergence, a third senior researcher was consulted or resolved by discussion. In the absence of mean and standard deviation (SD), we would transform them according to the corresponding formulae (22, 23). The extracted data of each included study contained the following: author, publication year, country of origin, age, intervention measures, treatment duration, and primary outcomes.

### Quality evaluation

The assessment of methodological quality in all included studies referred to the “risk of bias” evaluation tool produced by the Cochrane Collaboration Handbook for Systematic Reviews of Intervention (24). This was evaluated systematically and comprehensively in the following seven domains: the generation of random sequence, the concealment of allocation, blinding of participants and personnel, blinding of outcome assessors, completeness of outcome data, selective outcome reporting, and bias of other sources. All the studies were evaluated from the above domains from three levels of bias: “high risk”, “low risk”, and “unclear risk”. Discussion and consensus were reached *via* the third investigator while meeting discrepancies in quality assessment.

### Statistical analysis

Software Stata 17 and Review Manager (version 5.3) were used to log data and perform data analysis and quality assessment. The different effect measures were assigned to diverse variables, risk ratio (RR) for dichotomous variables and pooled mean difference (MD) for continuous variables. Furthermore, a 95% confidence interval (CI) was employed for both variables. In order to measure the heterogeneity of study results, I^2^ statistic was used. If heterogeneity (I^2^ > 50%, p < 0.05) was statistically significant, the random-effects model was applied for the analysis; otherwise, the fixed-effects model was selected. Simultaneously, subgroup analysis was conducted to explore the heterogeneity, and sensitivity analysis was performed to verify the robustness of our results. Finally, funnel plots and Egger’s test were performed for assessing publication bias when not less than 10 studies were brought into the analysis.

## Results

### Study selection and study characteristics

**Figure 1:**
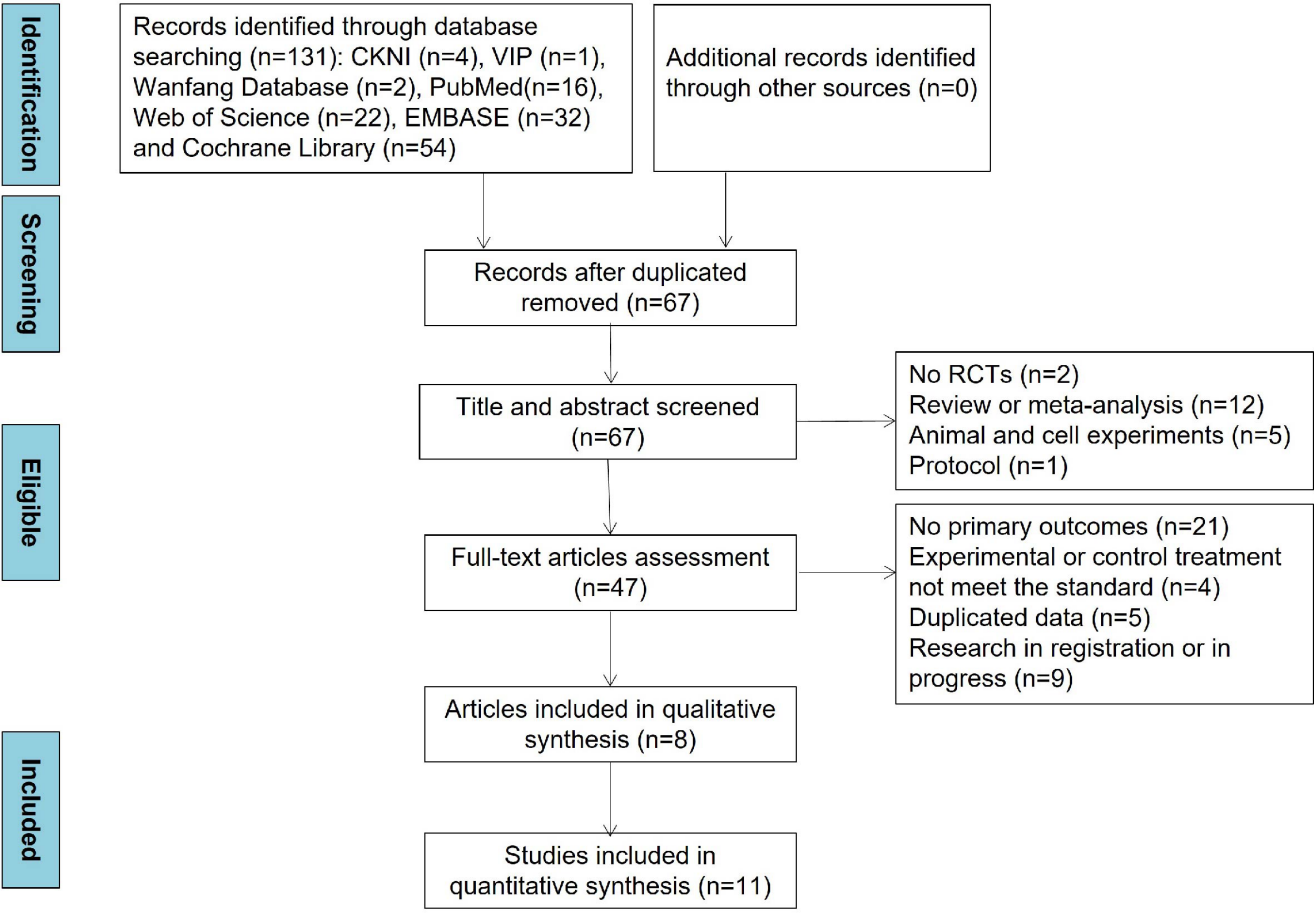
Flow diagram of literature screening.

The detailed study selection process and search results are presented in **Figure 1**. Through comprehensive database searching, a total of 131 articles were included. After duplicated articles were removed, 67 articles were assessed based on title and abstracts at first. Of these articles, 19 articles were excluded because of no RCTs (n = 2), review and meta-analysis (n = 12), animal and cell experiments (n = 5), and protocol (n = 1). Then, 39 articles were excluded for the reason of no primary outcomes (n = 21), irrelevant experimental or control treatment (n = 4), duplicated data (n = 5), and research in registration or progress (n = 9). Ultimately, 8 articles were included in qualitative synthesis and 11 studies in the quantitative synthesis. In addition, no medication treatment throughout the intervention periods changed in all included articles.

Eight (25–32) articles with 11 studies included were published from 2019 to 2022, and most of the patients were T2DM with NAFLD except for two studies (28, 31). A total of 686 randomized patients with a sample size ranging from 44 to 90 were included in this meta-analysis. Dapagliflozin and empagliflozin were the main intervention measures except for one trial (26) using ipragliflozin. Meanwhile, the treatment duration of SGLT2i varied from 12 to 24 weeks. The detailed characteristics of this meta-analysis are presented in **Table 1**.

**Table 1:** Basic characteristics of the included studies.

Study	Disease	Country	Sample size (E/C)	Male, %	Age, year	Treatment	Duration, weeks	Primary outcomes
Shimizu et al. (25)	T2DM+NAFLD	Japan	33/24	60	E: 56.2 ± 11.5 C: 57.1 ± 13.8	E: dapagliflozin 5 mg qd C: standard treatment	24	LSM, CAP
Taheri et al. (28)	NAFLD	Iran	43/47	56	E: 43.8 ± 9.7 C: 44.1 ± 9.3	E: empagliflozin 10 mg qd C: placebo	24	LSM, CAP
Lee et al. (31)	T2DM	China	30/30	60	E: 56.9 ± 10.7 C: 60.6 ± 7.03	E: dapagliflozin 10 mg qd + insulin C: sitagliptin 100 mg qd + insulin	24	LSM, CAP
Chehrehgosha a 2021 (29)	T2DM+NAFLD	Iran	17/34	49	E: 50.5 ± 8.4 C: 52.5 ± 7.9	E: empagliflozin 10 mg qd C: pioglitazone 30 mg qd	24	LSM, CAP
Chehrehgosha b 2021 (29)	T2DM+NAFLD	Iran	18/37	43	E: 50.5 ± 8.4 C: 51.8 ± 7.8	E: empagliflozin 10 mg qd C: placebo	24	LSM, CAP
Han et al. (26)	T2DM+NAFLD	Korea	29/15	61	E: 52.5 ± 10.3 C: 56.7 ± 11.8	E: ipragliflozin 50 mg qd + metformin + pioglitazone C: metformin + pioglitazone	24	CAP
Chu a 2022 (30)	T2DM+NAFLD	China	45/22	41	E: 46.73 ± 5.48 C: 46.29 ± 5.42	E: dapagliflozin 10 mg qd C: liraglutide (1 week: 0.6 mg; 2 weeks: 12 mg; 3–20 weeks: 1.8 mg qd)	20	LSM
Chu b 2022 (30)	T2DM+NAFLD	China	45/23	42	E: 46.79 ± 5.45 C: 46.29 ± 5.42	E: dapagliflozin 10 mg qd + liraglutide (1 week: 0.6 mg; 2 weeks: 12 mg; 3–20 weeks: 1.8 mg qd) C: Liraglutide (1 week: 0.6 mg; 2 weeks: 12 mg; 3–20 weeks: 1.8 mg qd)	20	LSM
Hu et al. (27)	T2DM+NAFLD	China	30/30	78	E: 48.9 ± 10.6 C: 52.1 ± 10.2	E: dapagliflozin 50 mg qd C: metformin 0.5 g tid	12	LSM, CAP
Tang (32)	T2DM+NAFLD	China	43/23	44	E: 49.44 ± 14.62 C: 47.36 ± 13.36	E: dapagliflozin 10 mg qd C: polyethylene glycol loxenatide 0.2 mg qw	12	LSM, CAP
Tang (32)	T2DM+NAFLD	China	45/23	47	E: 45.42 ± 9.58 C: 47.36 ± 13.36	E: dapagliflozin 10 mg qd + polyethylene glycol loxenatide 0.2 mg qw C: polyethylene glycol loxenatide 0.2 mg qw	12	LSM, CAP

E, experimental group; C, control group; w, week; qd, once daily; tid, three times a day; qw, once a week; T2DM, type 2 diabetes mellitus; NAFLD, non-alcoholic fatty liver disease; LSM, liver stiffness measurement; CAP, controlled attenuation parameter.

### Risk of bias and publication bias

**Figure 2:**
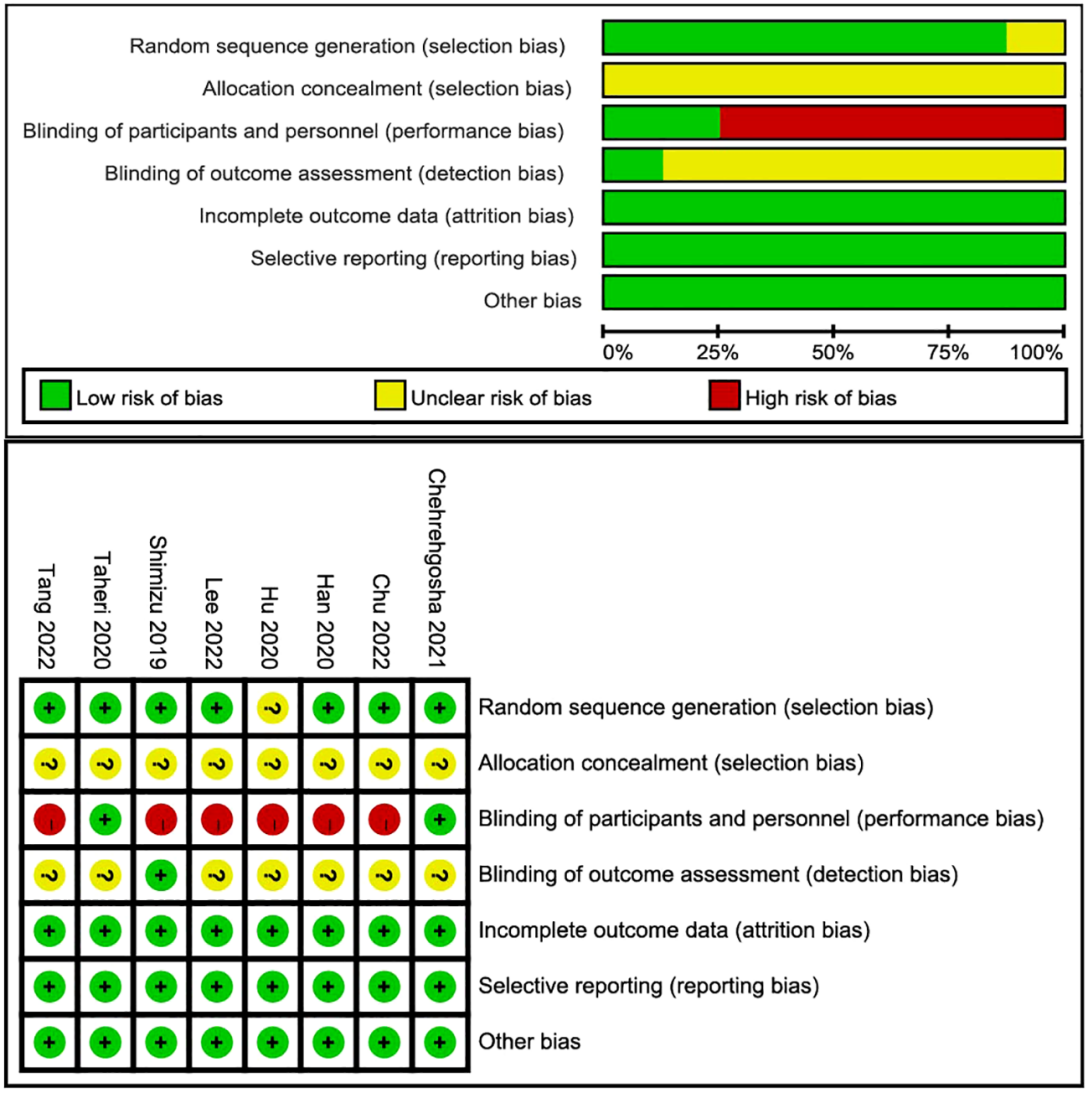
Risk-of-bias graph and risk-of-bias summary.

**Figure 3:**
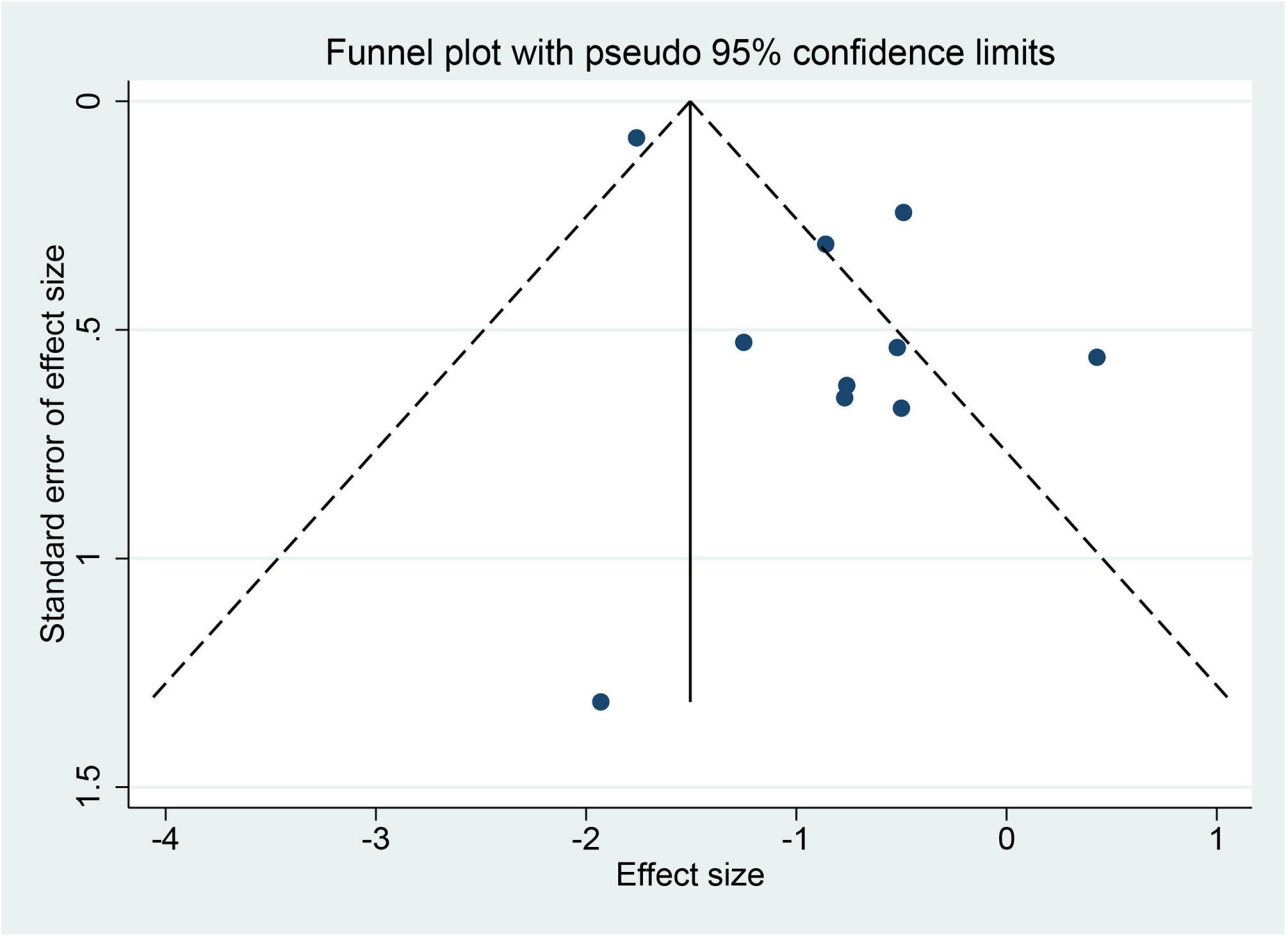
Funnel plot of liver stiffness measurement (LSM).

Among the eight articles, seven described the clear random sequence generation, and only one (27) merely mentioned “random”. None of the studies depicted the allocation concealment, so they ascribed to “unclear risk”. Two studies (28, 29) explicitly indicated blinding of participants and personnel, and the remaining was high risk. Only one (25) study pointed to the blinding of outcome assessment as straightforward. Attrition bias, reporting bias, and other biases were recognized as high risk. The risk-of-bias graph and risk-of-bias summary are shown in **Figure 2**. Because 10 studies recording LSM were included in this meta-analysis, asymmetry and sparsely distributed data were shown in funnel plots from visual inspection (**Figure 3** **)**, and Egger’s test verified this (p = 0.015), indicating that publication bias existed.

### Primary outcome: LSM and CAP

**Figure 4:**
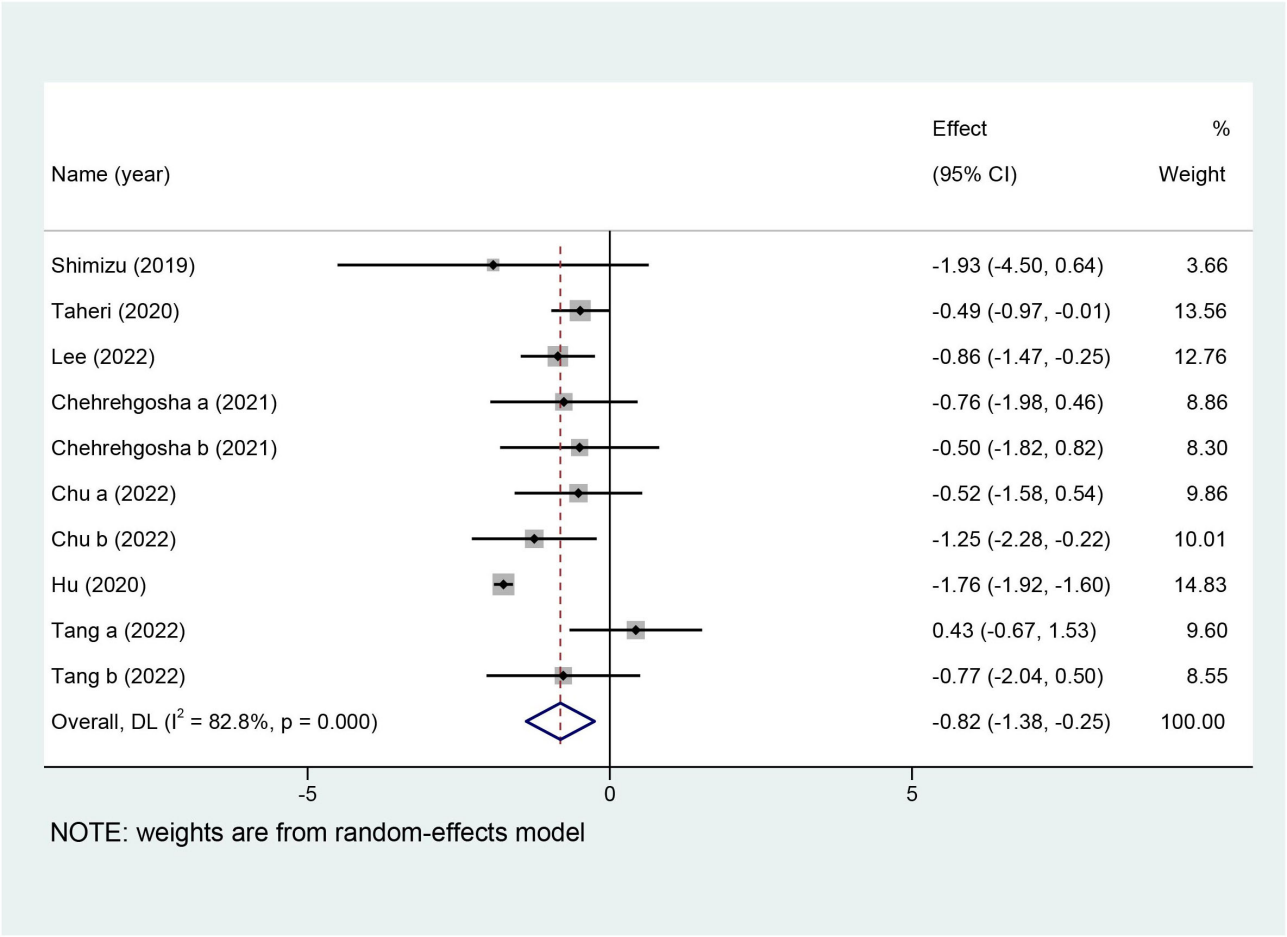
Forest plot of liver stiffness measurement (LSM).

**Figure 5:**
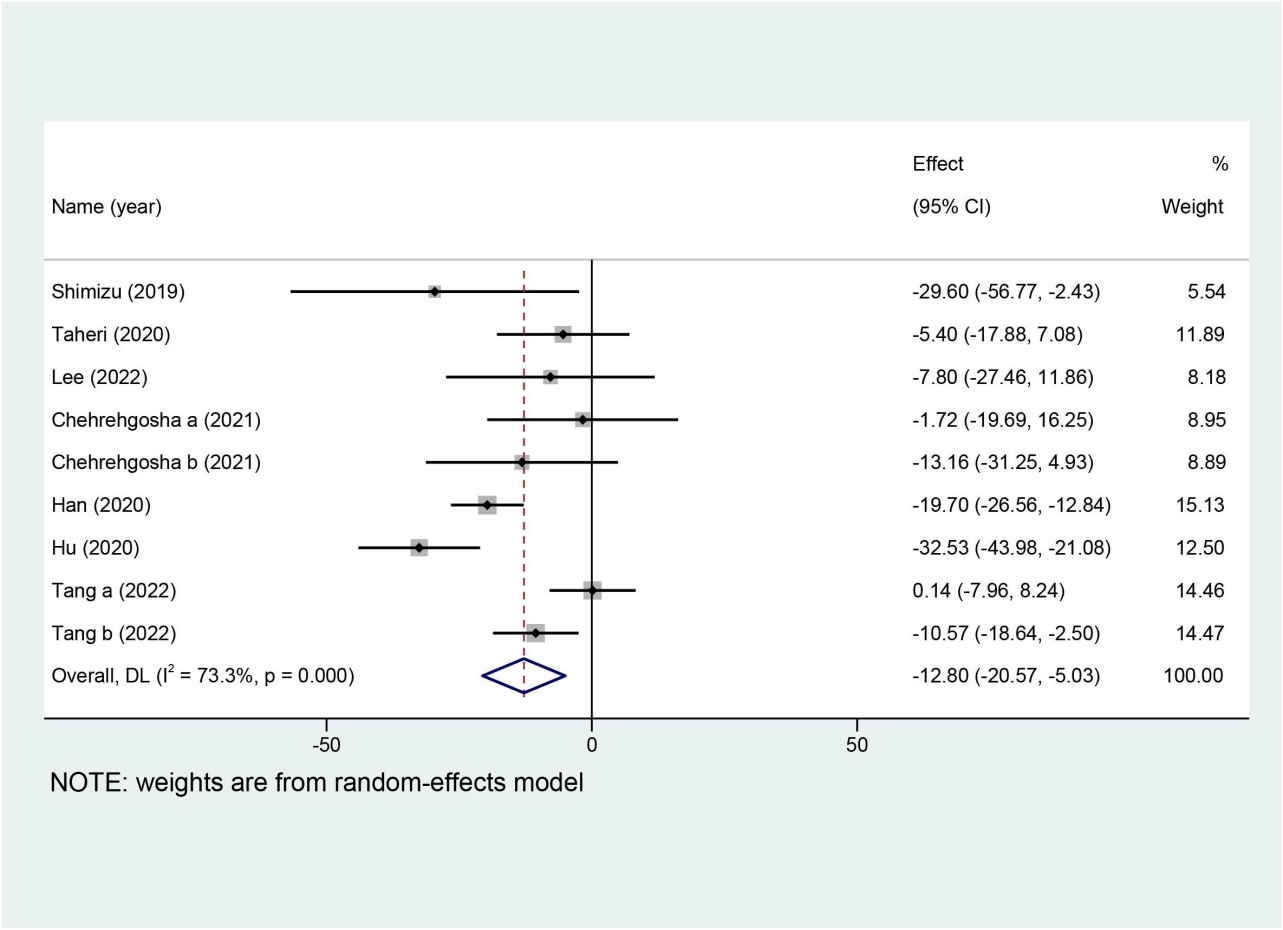
Forest plot of controlled attenuation parameter (CAP).

Ten studies with a total of 642 patients reported the effects of SGLT2i on LSM, and nine studies with 551 patients reported the effects of SGLT2i on CAP. Due to the high heterogeneity noted (I^2^ = 82.8%), a random-effects model was utilized to analyze the data. Overall effects in the z-test result were statistically significant [MD = −0.82, 95%CI (−1.38, −0.25), p = 0.005], indicating that SGLT2i significantly decreased LSM compared with the control group. For the outcome of CAP, a random-effects model was chosen because the collection in our meta-analysis presented obvious heterogeneity (I^2^ = 73.3%). SGLT2i could further decline the levels of CAP in comparison with the control group [MD = −12.80, 95%CI (−20.57, −5.03), p = 0.001]. Forest plots for the overall effects on the treatment of LSM and CAP are respectively shown in **Figures 4**, **5**.

### Sensitivity analysis

Sensitivity analyses were conducted to evaluate the robustness of our conclusions. As shown in **Supplementary Figures S1**, **S2**, the association between treatment with SGLT2i on LSM and CAP did not present considerable change after shifting out the study one by one, indicating that the pooled results were believable.

### Subgroup analysis

Due to high heterogeneity and sufficient reported studies noted in LSM and CAP, further subgroup analyses were conducted to investigate the sources of heterogeneity according to body mass index (BMI) (<30 and >30 kg/m^2^), treatment duration (≥24 and <24 weeks), age (>55 and <55 years), type of SGLT2i baseline of LSM (>7.3 and <7.3 kPa), and baseline of CAP (<292 and >292 dB/m). As shown in **Table 2**, the heterogeneity of patients with BMI >30 kg/m^2^, treatment duration ≥24 weeks, average age >55 years, treatment with empagliflozin, and baseline of LSM <7.3 kPa obviously decreased. However, treatment with SGLT2i did not lead to a significant decrease in the group of treatment duration of less than 24 weeks [MD = −0.85, 95%CI (−1.73, 0.03), p = 0.059] and baseline of CAP <292 dB/m [MD = −0.46, 95%CI (−1.26, 0.34), p = 0.257].

**Table 2:** Subgroup analyses of LSM based on BMI, treatment duration, age, type of SGLT2i, and baseline of LSM and CAP.

Criteria for grouping	Subgroups	n	MD (95%CI)	I^2^ (%)	Z	p
BMI	>30 kg/m^2^	3	−0.52 (−0.94, −0.10)	0	−2.437	0.015
	<30 kg/m^2^	7	−0.93 (−1.60, −0.26)	79.2	−2.723	0.006
Treatment duration	≥24 weeks	5	−0.65 (−1.00, −0.31)	0	−3.731	<0.001
	<24 weeks	5	−0.85 (−1.73, 0.03)	82.2	−1.891	0.059
Age	>55 years	2	−0.92 (−1.51, −0.32)	0	−3.017	0.003
	<55 years	8	−0.75 (−1.42, −0.09)	85.4	−2.220	0.026
Type of SGLT2i	Dapagliflozin	7	−0.93 (−1.60, −0.26)	79.2	−2.723	0.006
	Empagliflozin	3	−0.52 (−0.94, −0.10)	0	−2.437	0.015
Baseline of LSM	>7.3 kPa	6	−0.93 (−1.75, −0.12)	77.8	−2.239	0.025
	<7.3 kPa	4	−0.63 (−0.98, −0.28)	0	−3.567	<0.001
Baseline of CAP	>292 dB/m	5	−1.03 (−1.88, −0.18)	86.4	−2.376	0.018
	<292 dB/m	3	−0.46 (−1.26, 0.34)	51.7	−1.133	0.257

BMI, body mass index; n, number of studies; SGLT2i, sodium-glucose cotransporter 2 inhibitors; LSM, liver stiffness measurement; CAP, controlled attenuation parameter.

As detailed results of subgroup analyses of CAP are shown in **Table 3**, obviously reduced CAP levels were found in groups of treatment duration ≥24 weeks and treatment with ipragliflozin for the reason of declined heterogeneity and presence of significance in the same criteria for grouping. However, there was only one study included regarding ipragliflozin. Interestingly, although the heterogeneity of subgroup analyses significantly reduced, the patients in subgroup analyses of average age >55 years [MD = −16.60, 95%CI (−37.56, 4.36), p = 0.121], treatment with empagliflozin [MD = −6.38, 95%CI (−15.30, 2.54), p = 0.161], baseline of LSM <7.3 kPa [MD = −6.62, 95%CI (−14.74, 1.50), p = 0.110], and baseline of CAP <292 dB/m [MD = −5.56, 95%CI (−13.39, 2.26), p = 0.163] presented no evidence of benefit for CAP compared with the control group.

**Table 3:** Subgroup analyses of CAP based on BMI, treatment duration, age, type of SGLT2i, and baseline of LSM and CAP.

Criteria for grouping	Subgroups	n	WMD (95%CI)	I^2^ (%)	Z	p
BMI	>30 kg/m^2^	4	−11.71 (−20.97, −2.45)	51.7	−2.478	0.013
	<30 kg/m^2^	5	−14.75 (−27.81, −1.69)	82.4	−2.213	0.027
Treatment duration	≥24 weeks	6	−12.81 (−20.40, −5.22)	36.2	−3.309	0.001
	<24 weeks	3	−13.87 (−30.76, 3.03)	90.4	−1.608	0.108
Age	>55 years	2	−16.60 (−37.56, 4.36)	38.4	−1.552	0.121
	<55 years	7	−12.18 (−20.83, −3.53)	78.7	−2.759	0.006
Type of SGLT2i	Dapagliflozin	5	−14.75 (−27.81, −1.69)	82.4	−2.213	0.027
	Empagliflozin	3	−6.38 (−15.30, 2.54)	0	−1.402	0.161
	Ipragliflozin	1	−19.70 (−26.56, −12.84)	0	−5.628	<0.001
Baseline of LSM	>7.3 kPa	4	−16.34 (−31.65, −1.03)	86.8	−2.092	0.036
	<7.3 kPa	4	−6.62 (−14.74, 1.50)	0	−1.598	0.110
Baseline of CAP	>292 dB/m	6	−16.96 (−26.47, −7.46)	65.4	−3.499	<0.001
	<292 dB/m	3	−5.56 (−13.39, 2.26)	41.7	−1.394	0.163

BMI, body mass index; n, number of studies; SGLT2i, sodium-glucose cotransporter 2 inhibitors; LSM, liver stiffness measurement; CAP, controlled attenuation parameter.

## Discussion

Our meta-analysis found that SGLT2i was significantly associated with reduced LSM and CAP, although their heterogeneity was high. Therefore, subgroup analyses were conducted to reveal that treatment duration, age, type of SGLT2i, and baseline of LSM might be the sources of heterogeneity. In addition, BMI and baseline of CAP could be extra sources of heterogeneity in LSM and CAP respectively.

Fibrosis is the natural response to the injury. It plays a healing effect at an early stage, and if there is ongoing liver injury, fibrosis would be counterproductive with thick fibrous septae and distorted liver architecture (33). Different sizes of probes are used to generate low-frequency shear waves, the speed of which is quantitatively interpreted as LSM, presented as kPa (34). Meanwhile, assessed with liver biopsy, LSM is strongly associated with the stage of fibrosis (35). Results showed that treatment duration ≥24 weeks and baseline of CAP >292 dB/m might be more effective in lowering LSM than <24 weeks and <292 dB/m, respectively. On the one hand, existing high heterogeneity could not be ignored; on the other hand, insufficient treatment duration and no obvious change of CAP might not present statistical significance.

Hepatic steatosis is essential for diagnosing NAFLD, and in a steatosis liver, ultrasound energy is dissipated more rapidly. CAP measured by FibroScan is better than conventional abdominal ultrasound, the commonly used diagnostic method for liver steatosis, to identify a quantitative steatosis grading in favor of surveillance and treatment (36). p-Value on CAP in subgroup analyses of treatment duration <24 weeks, baseline of CAP <292 dB/m, age >55 years, treatment with empagliflozin, and baseline of LSM <7.3 kPa showed negative results, although the heterogeneity in the latter four groups significantly declined. Lack of sufficient treatment duration, older patients, and small changes in LSM and CAP might not obtain considerable effects, better absorption of medicinal effects, and statistical significance, respectively. In addition, a study (37) indicated that the diagnostic performance of FibroScan decreased when the fibrosis was in a mild state, which could also explain the lack of significant effect on CAP in the subgroup analysis of baseline of LSM <7.3 kPa. Empagliflozin and dapagliflozin both alleviated liver inflammation, suppressed hepatic lipogenesis, and attenuated hepatocellular injury (38–41); however, the obvious difference between them was unclear, and it was hard to explain why the latter was prior to the former on CAP. The possibility of the existence of high heterogeneity is still not ruled out.

In total, these negative results have been less studied, so it is a great possibility that the sample size is too small to be reliable. If the sample size increases, the results may be reversed. Nevertheless, we still have to keep in mind that the results should be interpreted cautiously.

The most intuitive discoveries of this meta-analysis were that SGLT2i was significantly correlated with reduced CAP and LSM. NAFLD is a major cause of cryptogenic cirrhosis (42), which can progress to cirrhosis, and the prevalence of advanced liver fibrosis in T2DM accompanied with NAFLD is 17% (78 million) (8). A growing consensus proposed that FibroScan could assess clinically significant fibrosis, and early intervention to prevent cirrhosis could be performed from a practical perspective (43–45). SGLT2i is a type of highly expressed membrane protein in the proximal tubules, the inhibitors of which can inhibit renal glucose reabsorption in the process of the glomerular filtration process and induce glycosuria (46). SGLT2i could decrease hepatic fat content and liver enzymes and improve body composition in contrast to other antihyperglycemic drugs, independent of insulin resistance, plasma glucose, and weight loss (19, 47). However, other investigations found that improvement in NAFLD is closely associated with body weight and plasma glucose (48, 49). Hence, it is yet known whether the positive effects of SGLT2i on hepatic fibrosis and steatosis are attributed to a direct effect of SGLT2i or adjustments of metabolic disturbance. Of note, there is no direct evidence demonstrating the role of SGLT2i on cirrhosis using LSM and CAP as outcomes.

To the best of our knowledge, this meta-analysis is the first to specifically focus on CAP and LSM to evaluate the efficacy of SGLT2i by using transient elastography. The meta-analysis by Wei et al. drew conclusions that SGLT2i could remarkably decline hepatic enzymes, liver proton density fat fraction, and visceral and subcutaneous fat area, but our research utilized transient elastography to intuitively explore liver conditions (19). The previous systematic review by Zhang et al. evaluated the effects of SGLT2i on hepatic fibrosis and steatosis, but they did not perform a quantitative analysis (13). Another meta-analysis merely included three articles accessing empagliflozin in comparison with placebo on NAFLD, and empagliflozin did not improve in CAP, LSM liver enzymes, and blood lipids (50). Relatively small samples and high heterogeneity may explain the contrasting conclusions. Furthermore, LSM and CAP as outcomes were included in evaluating the effects of SGLT2i, the results of which were negative, different from our results; however, the studies were few, no more than three, and had language restriction of English (18). Meanwhile, they used magnetic resonance imaging–proton density fat fraction or magnetic resonance spectroscopy to assess liver fat content, but they were limited due to the expensive equipment, which makes techniques hard to be popularized.

This meta-analysis still has some limitations that were almost inherent to the RCTs included. Firstly, no high-quality RCTs were included, where the evaluation of most detection bias and selection bias of all was unclear, and even some performance bias was high, which affected the credibility. Secondly, our meta-analysis had a language restriction, causing some selection bias. Thirdly, the sample size of included studies was less than 100 patients, so the results should be interpreted with caution. Fourthly, since there was a publication bias in the evaluation of LSM, serious attention should be paid to the results. Lastly, peroxisome proliferator-activated receptor agonists and glucagon-like peptide-1 receptor agonists also had positive effects in patients with NAFLD. We could not identify which antihyperglycemic drug was superior, so head-to-head RCTs are needed (15). Similarly, based on these, it is desirable that the findings should be interpreted with caution, and massive studies with allocation concealment, with blindness, are multi-centered, and with large sample sizes are warranted to be further established in the future. Despite the aforementioned limitations, this meta-analysis and systematic review still provides valuable information to assist us to understand the effects of SGLT2i on hepatic fibrosis and steatosis.

## Conclusions

This meta-analysis provided evidence for the efficacy of SGLT2i in reducing CAP and LSM, although high heterogeneity still existed. Therefore, we can conclude that SGLT2i could delay the progression of hepatic fibrosis and steatosis and be expected to be a specific drug in treating NAFLD. However, it is still necessary to endorse more randomized, double-blinded, multi-centered clinical trials with longer duration to evaluate SGLT2i on hepatic fibrosis and steatosis so that optimal decisions could be made by patients and clinicians together for proper clinical practice.

## Author contributions

PZ proposed the subject and designed the protocol for this systematic review. PZ, ZH and YT conducted literature screening and data extraction.YT and WX performed statistical analysis. PZ and YT drafted the manuscript. XZ and JY inspected all aspects of this systematic review. All authors contributed to the article and approved the submitted version.
