# Supplementary figures and images for: Effects of SGLT2 inhibitors on hepatic fibrosis and steatosis: A systematic review and meta-analysis
Source: Front Endocrinol (Lausanne). 2023 Mar 1;14:1144838. doi: 10.3389/fendo.2023.1144838 (PMC10014961; doi:10.3389/fendo.2023.1144838)

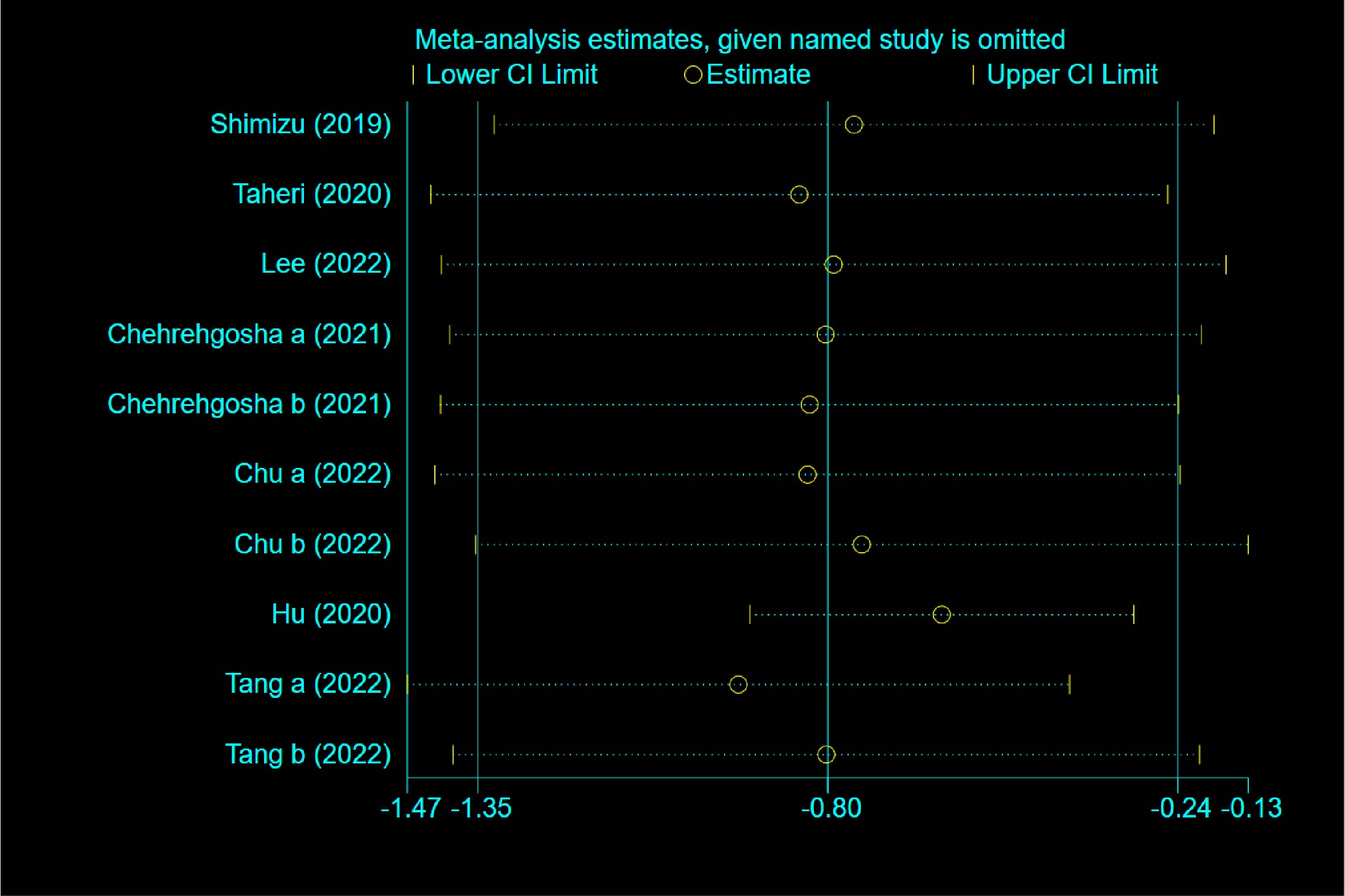

Supplement: Supplementary file 1 [file Image_1.jpeg]

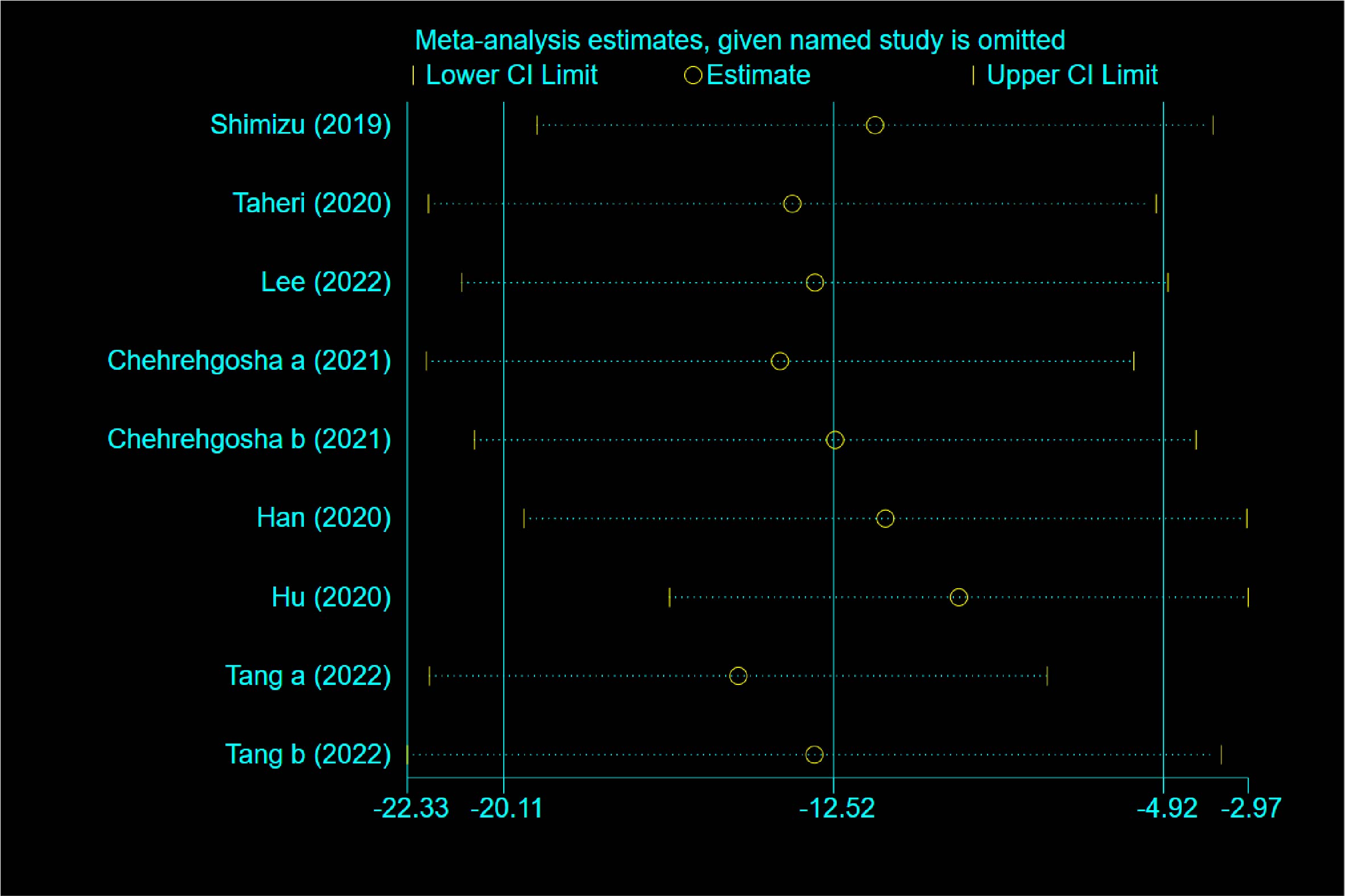

Supplement: Supplementary file 2 [file Image_2.jpeg]
