# Supplementary material for: Effects of SGLT2 inhibitors on hepatic fibrosis and steatosis: A systematic review and meta-analysis
Source: Front Endocrinol (Lausanne). 2023 Mar 1;14:1144838. doi: 10.3389/fendo.2023.1144838 (PMC10014961; doi:10.3389/fendo.2023.1144838)
Supplement: Supplementary file 3 [file Table_1.docx]

Table 1 Search strategy

| Database | Search strategy | Results | |
| --- | --- | --- | --- |
| 1)PubMed  (To November 30, 2022) | #1 | "canagliflozin"[Text Word] OR "empagliflozin"[Text Word] OR "dapagliflozin"[Text Word] OR "ertugliflozin"[Text Word] OR "sotagliflozin"[Text Word] OR "ipragliflozin"[Text Word] OR "luseogliflozin"[Text Word] OR "tofogliflozin"[Text Word] OR "remogliflozin"[Text Word] OR "bexagliflozin"[Text Word] OR "gliflozin"[Text Word] OR "Sodium-glucose cotransporter 2 inhibitor"[Text Word] OR "SGLT2"[Text Word] | 9017 |
|  | #2 | "transient elastography"[Text Word] OR "TE"[Text Word] OR "controlled attenuation parameter"[Text Word] OR "CAP"[Text Word] OR "liver stiffness"[Text Word] OR "LSM"[Text Word] OR "FibroScan"[Text Word] | 88325 |
|  | #3 | "random*"[Text Word] OR "blind*"[Text Word] | 1759904 |
|  | #4 | #1 AND #2 AND #3 | 16 |
| 2) Embase  (To November 30, 2022) | #1 | 'canagliflozin'/exp OR canagliflozin OR 'empagliflozin'/exp OR empagliflozin OR 'dapagliflozin'/exp OR dapagliflozin OR 'ertugliflozin'/exp OR ertugliflozin OR 'sotagliflozin'/exp OR sotagliflozin OR 'ipragliflozin'/exp OR ipragliflozin OR 'luseogliflozin'/exp OR luseogliflozin OR 'tofogliflozin'/exp OR tofogliflozin OR 'remogliflozin'/exp OR remogliflozin OR 'bexagliflozin'/exp OR bexagliflozin OR 'gliflozin'/exp OR gliflozin | 20644 |
|  | #2 | 'sodium-glucose cotransporter 2 inhibitor'/exp OR 'sodium-glucose cotransporter 2 inhibitor' OR sglt2 | 21707 |
|  | #3 | #1 OR #2 | 20427 |
|  | #4 | ('transient elastography':ti,ab,kw OR te:ti,ab,kw OR 'controlled attenuation parameter':ti,ab,kw OR cap:ti,ab,kw OR 'liver stiffness':ti,ab,kw OR lsm:ti,ab,kw) OR fibroscan:ti,ab,kw | 126179 |
|  | #5 | random* OR blind* | 2370599 |
|  | #6 | #3 OR #4 OR #5 | 32 |
| 3) Cochrane Library  (To November 30, 2022) | #1 | "canagliflozin"[All Text] OR "empagliflozin"[All Text] OR "dapagliflozin"[All Text] OR "ertugliflozin"[All Text] OR "sotagliflozin"[All Text] OR "ipragliflozin"[All Text] OR "luseogliflozin"[All Text] OR "tofogliflozin"[All Text] OR "remogliflozin"[All Text] OR "bexagliflozin"[All Text] OR "gliflozin"[All Text]OR "Sodium-glucose cotransporter 2 inhibitor"[All Text] OR "SGLT2"[All Text] | 4698 |
|  | #2 | "transient elastography"[All Text] OR "TE"[All Text] OR "controlled attenuation parameter"[All Text] OR "CAP"[All Text] OR "liver stiffness"[All Text] OR "LSM"[All Text] OR "FibroScan"[All Text] | 18376 |
|  | #3 | "random*"[All Text] OR "blind*"[All Text] | 1342018 |
|  | #4 | #1 AND #2 AND #3 | 54 |
| 4) Web of science  (To November 30, 2022) | #1 | **TS=(‘canagliflozin’ OR ‘empagliflozin’ OR ‘dapagliflozin’ OR ‘ertugliflozin’ OR ‘sotagliflozin’ OR ‘ipragliflozin’ OR ‘luseogliflozin’ OR ‘tofogliflozin’ OR ‘remogliflozin’‘tofogliflozin’ OR ‘bexagliflozin’‘tofogliflozin’ OR ‘gliflozin’ OR ‘Sodium-glucose cotransporter 2 inhibitor’ OR ‘SGLT2’)** | 15239 |
|  | #2 | **TS=('transient elastography' or 'TE' OR 'controlled attenuation parameter' OR 'CAP' OR 'liver stiffness' OR 'LSM' OR 'FibroScan')** | 306217 |
|  | #3 | **AB=('random*' OR 'blind*')** | 2317348 |
|  | #4 | #1 AND #2 AND #3 | 22 |
